# Saliva as a potential tool for cystic fibrosis diagnosis

**DOI:** 10.1186/1746-1596-8-46

**Published:** 2013-03-19

**Authors:** Aline Cristina Gonçalves, Fernando Augusto de Lima Marson, Regina Maria de Holanda Mendonça, José Dirceu Ribeiro, Antonio Fernando Ribeiro, Ilma Aparecida Paschoal, Carlos Emílio Levy

**Affiliations:** 1 Department of Pediatrics, Faculty of Medical Sciences, University of Campinas, P.O. Box: 6111, Campinas, SP 13081-970, Brazil; 2 Department of Genetics, Faculty of Medical Sciences, University of Campinas, P.O. Box: 6111, Campinas, SP, 13081-970, Brazil; 3 Department of Clinical Pathology, Faculty of Medical Sciences, University of Campinas, P.O. Box: 6111, Campinas, SP 13081-970, Brazil; 4 Boldrini Children's Center, Campinas, Brazil; 5 Department of Medical Clinics, Faculty of Medical Sciences, University of Campinas, P.O. Box: 6111, Campinas, SP, 13081-970, Brazil; 6 Department of Clinical Pathology, Alexander Fleming Street 105, FCM12, Second floor, Barão Geraldo, Campinas, SP, 13083-881, Brazil

**Keywords:** Saliva, Ion concentration, Cystic fibrosis, Lung disease, CFTR

## Abstract

**Background:**

Saliva and sweat are modified by cystic fibrosis (CF). In both cases the chloride and sodium ion concentrations for healthy subjects and CF patients differ, this representing a possible alternative tool for CF diagnosis. In this context, the aim of this study was to compare the concentrations of these ions in saliva samples taken from CF patients and healthy subjects.

**Methods:**

A case–control study was carried out at a university CF center, in which the saliva samples were analyzed on an ABL 835 Radiometer^®^ to determine the ion concentration.

**Results:**

For the CF patients (n = 80) the values for the biochemical parameters of chloride, potassium and sodium ion concentration were higher (p < 0.009) and the volume and pH of the saliva were lower than in the case of healthy subjects (p < 0.009). For the healthy subjects group (n = 84) versus CF patients, according to the ROC curve, the values for sodium were: cutoff: 13.5 mmol/L, sensitivity: 73.4%, specificity: 70.6%; and for chloride: cutoff: 20 mmol/L, sensitivity: 68.1%, specificity: 72.9%.

**Conclusions:**

The chloride and sodium concentrations in the saliva samples were higher for CF patients in comparison with healthy subjects. Thus, saliva as a tool for CF diagnosis can be considered a new challenge, and a population study including patients in all age classes needs to be performed, in different countries over the world, to extend the database to include a broad spectrum of information in order to identify normal ion concentration ranges for CF patients according to age, genotype and environment.

**Virtual Slides:**

The virtual slide(s) for this article can be found here: http://www.diagnosticpathology.diagnomx.eu/vs/2614233148750145

## Background

Cystic fibrosis (CF), the most common genetic disease in Caucasians, is a hereditary exocrinopathy with a wide range of clinical and genetic variants [1].

Paul di Sant'Agnese et al. [2] investigated the ionic composition of sweat in CF patients and found higher concentrations of chloride, sodium and potassium in comparison to healthy subjects. To decrease the difficulties associated with obtaining sufficient quantities of sweat to determine the concentration of electrolytes, Gibson and Cook [3] proposed using the cholinergic stimulation technique on a small area of skin via pilocarpine iontophoresis. The quantitative analysis of sweat chloride levels is currently applied as a discriminatory test for CF diagnosis [4].

The cystic fibrosis transmembrane conductance regulator (CFTR) protein forms a channel which allows chloride ions to cross the cell membrane of epithelial cells in both directions depending on the electrochemical gradient, this being important in the production of airway surface liquid, exocrine pancreatic secretion and sweat [5].

Sweat in healthy subjects is hypotonic in relation to the extracellular fluid to ensure an effective heat loss which is essential to maintaining the proper body temperature. In the sweat gland acini, the primary gland secretion has an ionic composition similar to that of interstitial fluid. The primary secretion of sweat glands, when passing through glandular ducts, loses chloride and sodium ions, which are absorbed by the CFTR and sodium (ENaC) channel, respectively. The ion absorption without corresponding water absorption leads to hypotonic sweat [6]. The mechanism of saliva production has similarities with that of sweat production.

Saliva is a secretion which is produced by four glands: parotid (serous secretion), oral (mucous secretion) and submandibular and sublingual (serous and mucous secretion). The primary secretion in the acinus of the glands has an ion concentration similar to those of the plasma and interstitial fluid. Along the ducts, the absorption of specific ions and secretion and/or passive movement in opposite directions reduces the levels of sodium and chloride ions and increases those of bicarbonate and potassium ions.

The involvement of CFTR and ENaC in the ducts of salivary glands has been demonstrated in homozygous mice for the F508del mutation [6]. Therefore, it can be hypothesized that these channels play a role in ion absorption in salivary gland ducts, similarly to sweat glands.

Iontophoresis as a stimulus for the production of sweat presents difficulties associated with: (i) a low amount of sweat, (ii) equipment availability, (iii) child immobilization and stress, (iv) obtaining the amount of sweat required to repeat the test.

Due to the drawbacks related to implementing this technique, other diagnostic possibilities have been sought and in this regard the biochemical parameters in saliva represent a potential tool for CF diagnosis. The collection method for saliva is simple and noninvasive and the donation process is associated with minimal stress allowing multiple collections to be performed. Oral fluid sampling is safe for both the operator and patient, and involves easy and low-cost storage [7,8]. Particularly in the case of CF, the use of saliva as a diagnostic test could bring benefits, considering that saliva is easier to collect than sweat [9].

In this context, studies on the effects of CF on the functioning of salivary glands have produced conflicting results. Also, the parameters considered, measurement methods used and conditions applied in the obtainment of saliva differ, as shown in Table 1, making it difficult to compare the results.

**Table 1:** Comparison of volume, pH and biochemical parameters of saliva in cystic fibrosis patients and healthy controls

**Parameter**	**CF patients (n=80)**		**Healthy subjects (n=84)**		p-value^***^	**p-value corrected by Bonferroni test**
	**Mean ± SD (95% CI)**	**Median**	**Mean ± SD (95% CI)**	**Median**		
Calcium*	2.03± 0.99 (0.30 – 4.65)	1.85	2.15 ± 2.79 (0.71 – 23)	1.61	0.114	1
Glucose*	13.02 ± 18.69 (3 – 147)	8	9.25± 3.56 (3 – 20)	9.5	0.932	1
Lactate*	0.91 ± 0,89 (0.2 – 5.6)	0.7	0.81± 0.74 (0.1 – 4.3)	0.6	0.463	1
Chloride*	27.82 ± 13.47 (12 – 89)	26	18.04 ± 8.3 (8 – 43)	16	**<0.001**	**<0.009**
Volume**	0.63 ± 0.3 (0.1– 1.5)	0.6	0.8 ± 0.5 (0 – 2)	1	**<0.001**	**<0.009**
**Ph**	6.86 ± 0.45 (6.27 – 7.8)	6.83	7.17 ± 0,44 (6.3 – 7.85)	7.2	**<0.001**	**<0.009**
Potassium*	19.66± 3.25 (10.8 – 24.8)	19.55	16.82 ± 3.22 (6.5 -24)	17.20	**<0.001**	**<0.009**
Sodium*	19.90 ± 9.3 (8 – 48)	17.5	12.26 ± 4.31 (7 – 29)	11	**<0.001**	**<0.009**
Bicarbonate*	5.53 ± 3.98 (0 – 22)	3	3.58 ± 3.98 (0 -16)	1.95	0.051	0.459

CF – Cystic Fibrosis, CI – Confidential interval, n – number of patients, SD – Standard Deviation.

* mmol/L.

** N for volume is 68 in CF patients and 64 in healthy subjects, unlike the other parameters.

*** The Statistical analyses were performed by Mann-Whitney test. The positive p-value is in bold.

In this context, the aim of this study was to compare the pH, volume of saliva and concentrations of bicarbonate, sodium, chloride, potassium, glucose, lactate and calcium in saliva samples taken from CF patients and healthy subjects, with a view to providing an alternative tool for CF diagnosis.

## Methods

An analytical observational study (case–control) was carried out for which individuals with or without the disease (cystic fibrosis) were selected. The **case group** (n = 80) was comprised of patients with CF at the Unicamp Teaching Hospital and the **control group** (n = 84) was comprised of healthy individuals recruited from a school (from 04 to 18 years old) and a university. The characterization according to age for both groups is shown in Table 2.

**Table 2:** Sample characterization according to age (years)

**Parameter**	**CF group**	**Control group**
No. of Patients	80	84
Mean	12.38	18.2
Median	12.0	17.0
Std. Deviation	7.1	5.24
Minimum	4.0	5.0
Maximum	34.0	28.0

CF – Cystic Fibrosis. There was no statistically significant difference between groups.

The statistical analysis was performed applying the Mann–Whitney test. The positive p-value is in bold.

The subjects of the control group were all from the same geographical region, were not taking any medications of continuous use and did not take any medicine during the saliva collection period.

In the case of the CF patients the diagnosis was carried out considering two chloride concentrations in sweat greater than or equal to 60 mEq/L and/or in the genetic study positive results for two CFTR gene mutations.

The saliva collection was performed after rinsing of the mouth with water for one minute to eliminate contamination and stimulate the salivary glands. The collection took place at the same time of the day (afternoon) for both the case group and control group to avoid possible physiological interferences.

The saliva samples were collected with a cotton swab - Salivet^®^ (Sardest-Germany - http://www.sarstedt.com) by chewing sterile cotton rolls for one minute. The samples were immediately centrifuged at 1800 rpm for 15 minutes after the collection and the saliva volume was measured.

Bicarbonate, pH, sodium, chloride, potassium, glucose, calcium and lactate concentrations were determined on an ABL gasometer (model 835, Radiometer^®^, Denmark) using 20 μL of saliva by gasometric analysis using an ion-selective electrode (http://www.radiometer.com).

The project was approved by the University Ethics Committee (#157/2010) and all patients and/or their guardians gave their informed consent.

### Statistical analysis

The calculation of the sample size using the program GPower^®^, version 3.1.2 [10] applying the Mann–Whitney Test, indicated that 67 individuals were needed for each group, considering a sample power of 80% and α = 0.05. The sample power was previously calculated by the statistics sector of the university.

The Mann–Whitney test was used to evaluate the difference between the results for the biochemical parameters obtained for the two groups (CF patients and healthy subjects), with a 5% significance level. The Receiver Operating Characteristic (ROC) curve was constructed to measure the sensitivity and specificity of chloride and sodium secretion as a tool for CF diagnosis.

The Statistical Package for the Social Sciences^®^ version 17 (SPSS) was used for the statistical analysis [11].

To avoid spurious data caused by the performance of multiple tests [12], the significance level (α) was adjusted using the Bonferroni correction method (α corrected = 0.05/number of tests).

## Results

The study comprised 164 subjects (46.99% male and 53.01% female), with a mean age of 13.3 years (±6.65). The mean age of the CF patients (n = 80) was 13.04 years (±7.27) and that of the healthy subjects (n = 84) was 13.56 years (±6.03 years). There was no statistically significant difference between the mean ages of the CF patients and healthy subjects (p = 0.297).

**Figure 1:**
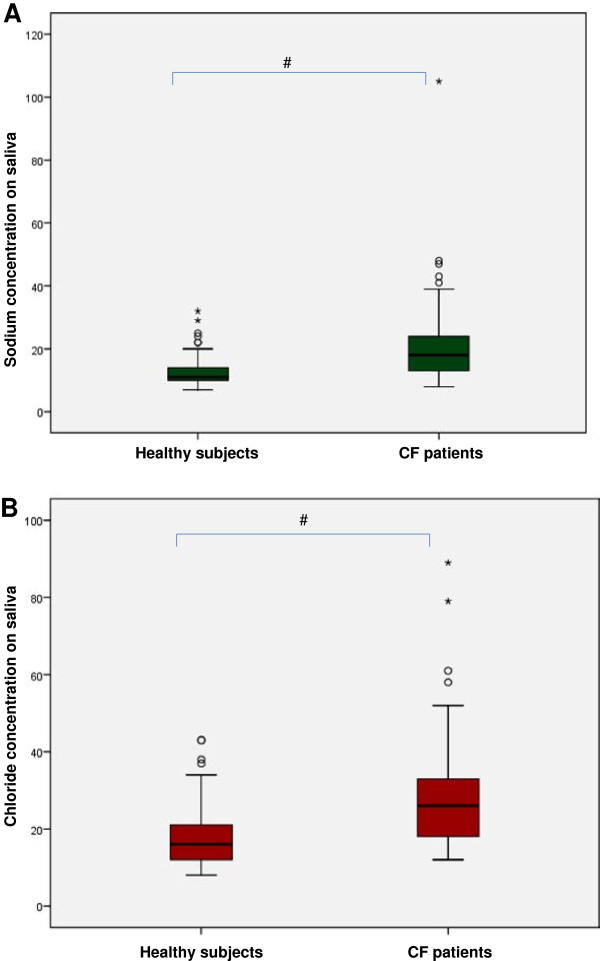
**Box plot of levels of sodium and chloride. A**. Comparison of sodium concentrations in saliva of healthy subjects and CF patients, * p < 0.009. **B**. Comparison of chloride concentrations in saliva of healthy subjects and CF patients, * p < 0.009.

The saliva markers and their distribution among groups (CF patients and healthy subjects) can be seen in Table 1. The chloride, potassium and sodium concentrations were higher in CF patients when compared with health subjects. The volume and pH of the saliva were lower for CF patients when compared with healthy subjects. In Figure 1, a box plot shows the sodium and chloride concentrations in the saliva (p < 0.009).

**Figure 2:**
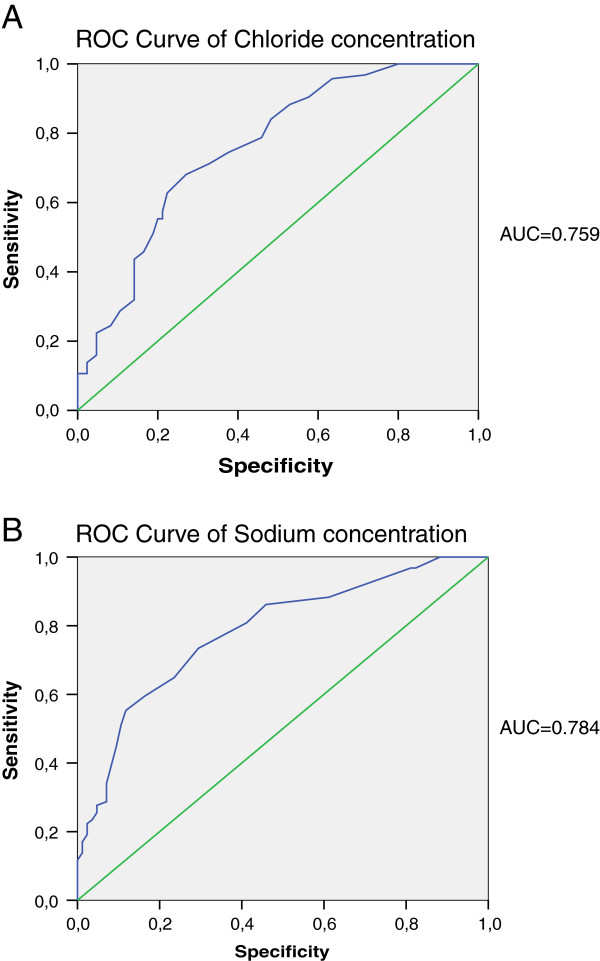
**ROC curves for chloride and sodium. A**. ROC curve for chloride concentration. Cutoff: 13.5mmol/l, sensitivity: 73.4%, specificity: 70.6%; **B**. ROC curve for sodium concentration. Cutoff: 20mmol/l, sensitivity: 68.1%, specificity: 72.9%.

The ROC curve compared the healthy subjects versus CF patients and the values for sodium were: cutoff: 13.5 mmol/L, sensitivity: 73.4%, specificity: 70.6%; and for chloride: cutoff: 20mmol/L, sensitivity: 68.1%, specificity: 72.9% (Figure 2).

## Discussion

The saliva composition can be affected by some diseases and thus can be used as a diagnostic tool [21]. In CF, the ion concentrations (sodium and chloride) in the sweat and saliva are altered. Although, the sweat test is a ‘gold standard’ tool to CF diagnosis, several problems are associated with this examination, such as: (i) lack of equipment, (ii) high cost, (iii) lack of professional specialists, (iv) difficult collection, (v) processing for chloride and sodium determination is not carried out at the same time or with the same method. In this context, there is a need for an alternative tool for CF diagnosis.

Results obtained in previous studies have indicated that assessment of the salivary profile, particularly of sodium and chloride concentrations, is important, suggesting that this approach could be used as a tool for CF diagnosis [21]. The results reported in the literature are difficult to compare for the following reasons: (i) methods used for the saliva collection and ion concentration determination differ; (ii) different patient inclusion criteria are applied; and (iii) the study populations are small. In this context, this study involved a larger population in comparison with others, and a new technique was used to determine the ion concentrations. It is also important to note the unprecedented use of equipment for blood gas analysis and the assessment of saliva biochemical markers.

Only eight studies on the biochemical parameters of saliva collected from CF patients could be found in the literature, involving different saliva sampling and ion concentration measurement methods, as shown in Table 3. Since 1996 no studies on saliva as a diagnostic tool for CF have been carried out. In three studies, atomic absorption spectrometry was used to measure the biochemical parameters. In one of these Jimenez-Reyes and Sanchez-Aguirre [20] evaluated the levels of chloride and sodium in the saliva of nine patients with CF and nine control subjects, and found differences between the groups, with an increase in the concentration of sodium in the CF group, as we observed in the study reported herein. In a study by Blomfield et al. [18], atomic absorption spectrometry was also used for the saliva assessment. After using 10% citric acid to stimulate saliva production in 63 individuals (35 with CF), the authors observed statistically significant differences in the values for both chloride and sodium on comparing the results for the two groups, as also reported herein. In another study, Wiesmann et al. [16] did not find statistically significant differences in the sodium results for the CF and control groups. Although the patients involved in these studies had ages similar to those of our study reported herein, the different technique used does not allow reliable comparison with our results.

**Table 3:** Literature data on sodium and chloride concentrations in saliva of patients with cystic fibrosis (CF)

**Researchers**	**Date**	**CFG°**	CG*	**Method**	**Results**
Chernick et al. [13]	1961	12	12	Flame photometry/titration	High level of potassium and low levels of sodium and chloride in patients with CF
Marmar et al. [14]	1966	12	13	Flame photometry/titration	High levels of sodium and chloride in CF patients
Lawson et al. [15]	1967	5	50	Electrode selective	High level of sodium in CF patients
Wiesmann et al. [16]	1970	23	12	Atomic absorption spectrophotometry	No significant differences in sodium level
Fritz et al. [17]	1972	11	0	Unquoted	High levels of chloride and sodium for saliva flow in CF patients
Blomfield et al. [18]	1973	35	28	Atomic absorption spectrophotometry	High levels of chloride and sodium in CF patients
Kollberg et al. [19]	1982	9	11	Flame-emission photometry	High level of sodium in CF patients
Jimenez-Reyes & Sanches-Aguirre [20]	1996	9	9	Atomic absorption spectrophotometry	High level of sodium in CF patients

°CFG: cystic fibrosis group; *CG: control group. Databases of Pubmed, Scielo, Medline and HighWire Stanford University, in the period of 1951 to 2011 were searched. The keywords were: biochemical parameters of saliva and cystic fibrosis.

Also, in agreement with our observations, in studies carried out by Marmar et al. [14] and Blomfield et al. [18] the chloride concentration in the saliva was high for CF patients in comparison with healthy subjects.

Chernick et al. [13] evaluated the saliva of 24 subjects divided into two groups: CF patients (n = 12) and healthy subjects (n = 12). The levels of sodium and potassium were analyzed by the flame-photometry technique and the chloride concentration by titration. The CF patients had lower levels of sodium and chloride, in contrast to the results obtained in our study. However, the potassium level in the CF group was higher than that of the control group, as we also found in our study.

It is noteworthy that in the present study no chemical was used to stimulate salivary flow, in contrast with most other studies on salivary parameters in CF patients. This procedure may alter the volume, pH and biochemical parameters of saliva. According to Catalán et al. [6], studies conducted to evaluate the effect of CF on the functioning of the salivary glands have produced conflicting results. Blomfield et al. [18,22] demonstrated that CF had no influence on the salivary flow of the patients. Marmar et al. [14], on evaluating 12 patients with CF and 13 healthy children, found that the volume of saliva was higher in CF patients at all times after salivary stimulation. However, in our study, we found that the salivary flow is decreased in the CF group, consistent with the findings of other studies cited herein [23,24]. Kollberg et al. [19] evaluated nine patients with CF and 11 control subjects and observed reduced salivary flow in CF patients. Ceder et al. [23] evaluated 12 patients, aged 8–16 years, before and after stimulation with 2% citric acid. The authors observed a reduction in the salivary flow of CF patients when compared with the control group, both before and after salivary stimulation. In these studies, the authors concluded that the results suggest a primary defect related to the disease or a secondary defect due to the destruction of the glandular parenchyma. Pedersen et al. [24], in a review of salivary functions, cited several causes in relation to hyposalivation including medications, autoimmune disease, endocrine disorders, genetic disorders (including CF), malnutrition and infection. Previous studies showed conflicting results regarding the changes in salivary flow in CF, but in these studies the number of subjects was too small to draw decisive conclusions on the subject, while the results reported herein suggest a more consistent finding of hyposalivation.

In this study, the mean pH of the saliva of the CF patients was lower than that of the healthy subjects. In this regard, we could not find another study considering the pH of saliva collected from CF patients in the literature.

Although the results obtained suggest that saliva composition is a possible tool for CF diagnosis, further studies need to be carried out to evaluate this hypothesis, given that the methods for assessing salivary parameters differ, which hinders comparisons. In relation to the limitations of this study, the difficulty associated with obtaining voluntary saliva from children under 7 years old should be highlighted. However, as previously mentioned, it is worth noting that saliva collection is a safe, noninvasive, simple and inexpensive method, which does not require dermal puncture and can be collected repeatedly without discomfort [25]. Thus, the evaluation of salivary composition may lead to the establishment of an alternative to the sweat test in the future, which is easier to conduct and more effective. Some biochemical parameters of saliva, such as potassium, chloride, sodium, pH and volume appear to be influenced by CF. Further studies are needed to confirm the usefulness of the evaluation of salivary parameters as a complementary method for diagnosing this disease.

## Conclusions

The results of this study indicated that saliva cannot be used as a diagnosis tool for CF. Nevertheless, with the application of other methods to identify the ion concentration of saliva, in studies based on a sufficiently large population, and by performing the comparison considering the saliva and sweat tests at the same time, new insights could be gained regarding the use of saliva as an important CF diagnosis tool.

## Abbreviations

CF: Cystic Fibrosis; CFTR: Cystic fibrosis transmembrane conductance regulator; ENaC: Epithelial Na + channel; Rpm: Revolutions per minute; ROC: Receiver Operating Characteristic.

## Competing interests

The authors declare that they have no competing interests.

## Authors' contributions

ACG/CEL: Responsible for collecting and analyzing samples, inputting test results, data analysis, comparison of results with others published in the literature, publication of results and writing of the article. RMHM/IAP/FALM/AFR/JDR: Collaboration in data analysis, comparison of results with others published in the literature, publication of results and writing of the article. All authors read and approved the final manuscript.
